# Supplementary material for: Local crystallization inside the polymer electrolyte for lithium metal batteries observed by operando nanofocus WAXS
Source: Nat Commun. 2025 Oct 8;16:8958. doi: 10.1038/s41467-025-64736-w (PMC12508461; doi:10.1038/s41467-025-64736-w)
Supplement: Supplementary file 1 — Supplementary Information [file 41467_2025_64736_MOESM1_ESM.pdf]

# Supplementary Information

# Local crystallization inside the polymer electrolyte for lithium metal batteries observed by *operando* nanofocus WAXS

*Fabian A.C. Apfelbeck*[1], *Gilles E. Wittmann*[2], *Morgan P. Le Dû*[1], *Lyuyang Cheng*[1], *Yuxin Liang*[1], *Yingying Yan*[1], *Anton Davydok*[3], *Christina Krywka*[3], *and Peter Müller-Buschbaum*[1]*

[1]TUM School of Natural Sciences, Department of Physics, Chair for Functional Materials, Technical University of Munich, James-Franck-Str. 1, 85748 Garching, Germany

[2]Heinz Maier-Leibnitz-Zentrum (MLZ), Technical University of Munich, Lichtenbergstraße 1, 85748 Garching, Germany

[3]Helmholtz-Zentrum Hereon, Max-Planck-Str. 1, 21502 Geesthacht, Germany

*Corresponding author. **E-Mail: muellerb@ph.tum.de**

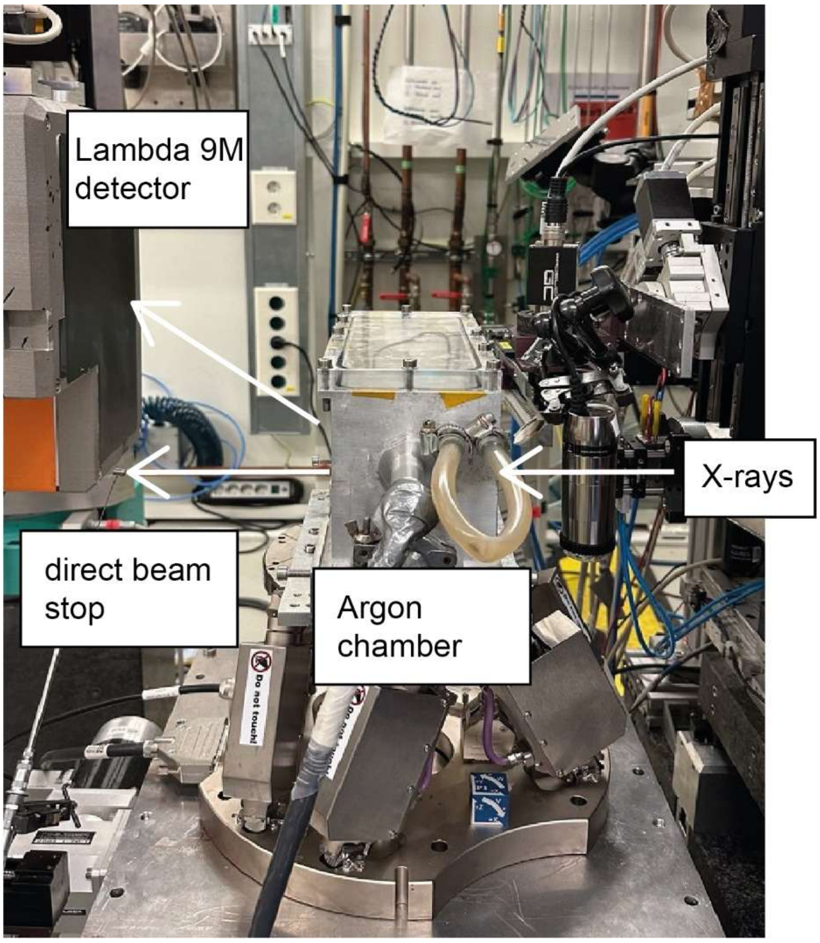


**Supplementary Figure 1**. Experimental GIWAXS setup at DESY for determining the crystalline structure of the pristine polymer electrolyte in a controlled argon atmosphere. The corresponding result of this measurement is given in Fig. 2a.

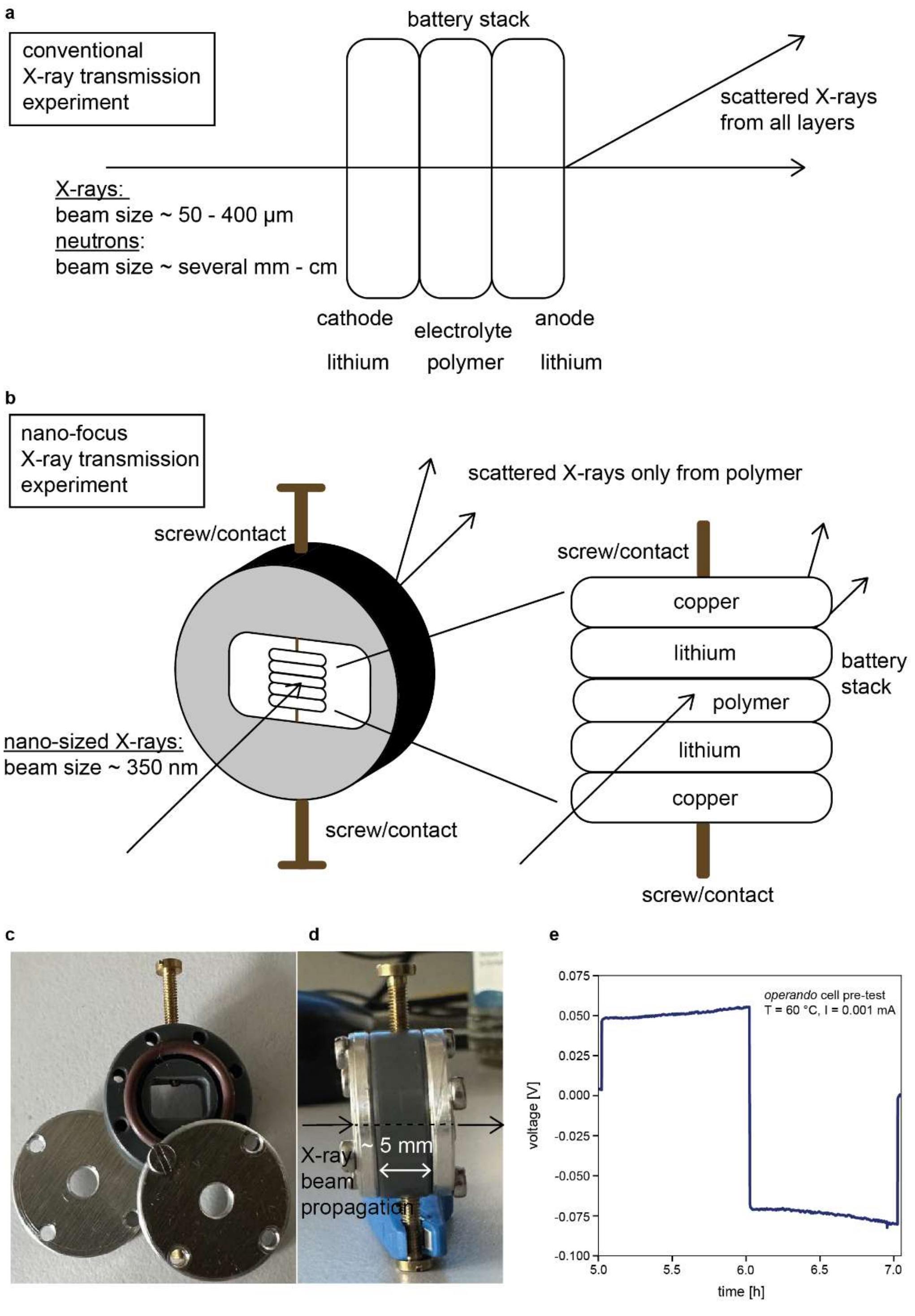


**Supplementary Figure 2**. **a** Schematic of a conventional X-ray transmission experiment with the X-ray beam being oriented parallel to the normal of the battery. **b** Schematic of the nano-focus X-ray transmission experiment with the X-ray beam being oriented parallel to the battery electrodes. **c** Photograph of the opened nano-focus WAXS battery cell, **d** side view of the closed battery cell, and **e** electrochemical pre-test of the nano-focus WAXS battery cell. Source data are provided as a Source Data file.

The beam size of X-rays and neutrons is typically quite large: for X-rays, ~ 50 - 400 µm, and neutrons, ~ several mm - cm. This makes a local investigation of one specific battery layer (for example, the polymer electrolyte) with scattering experiments very difficult and practically impossible. Therefore, in almost every battery *operando* transmission scattering experiment, the beam direction is parallel to the normal of the battery stack and hence every layer, meaning both electrodes and the electrolyte, are penetrated by X-rays or neutrons. Consequently, the resulting scattering signal contains information about all penetrated layers, and potential structure changes cannot be clearly assigned to one specific layer (for example, the polymer electrolyte). The specific innovation of using a nano-sized X-ray beam is that with the extremely small beam size of ~ 330 nm x 330 nm, the beam direction can be oriented parallel to the electrodes, and the aforementioned issue can be avoided. The X-ray beam only penetrates the polymer layer, and possible structure changes can be clearly assigned to the polymer layer.

The custom-made cell is electrochemically tested with a Li metal/polymer electrolyte/Li metal configuration at T = 60 °C, and the result is shown in Supplementary Fig. 2e. The slight asymmetric voltage behavior might originate from a different pressure from the screws, which is set by hand. Nevertheless, the two plating and stripping plateaus verify the electrochemical functionality of the cell.

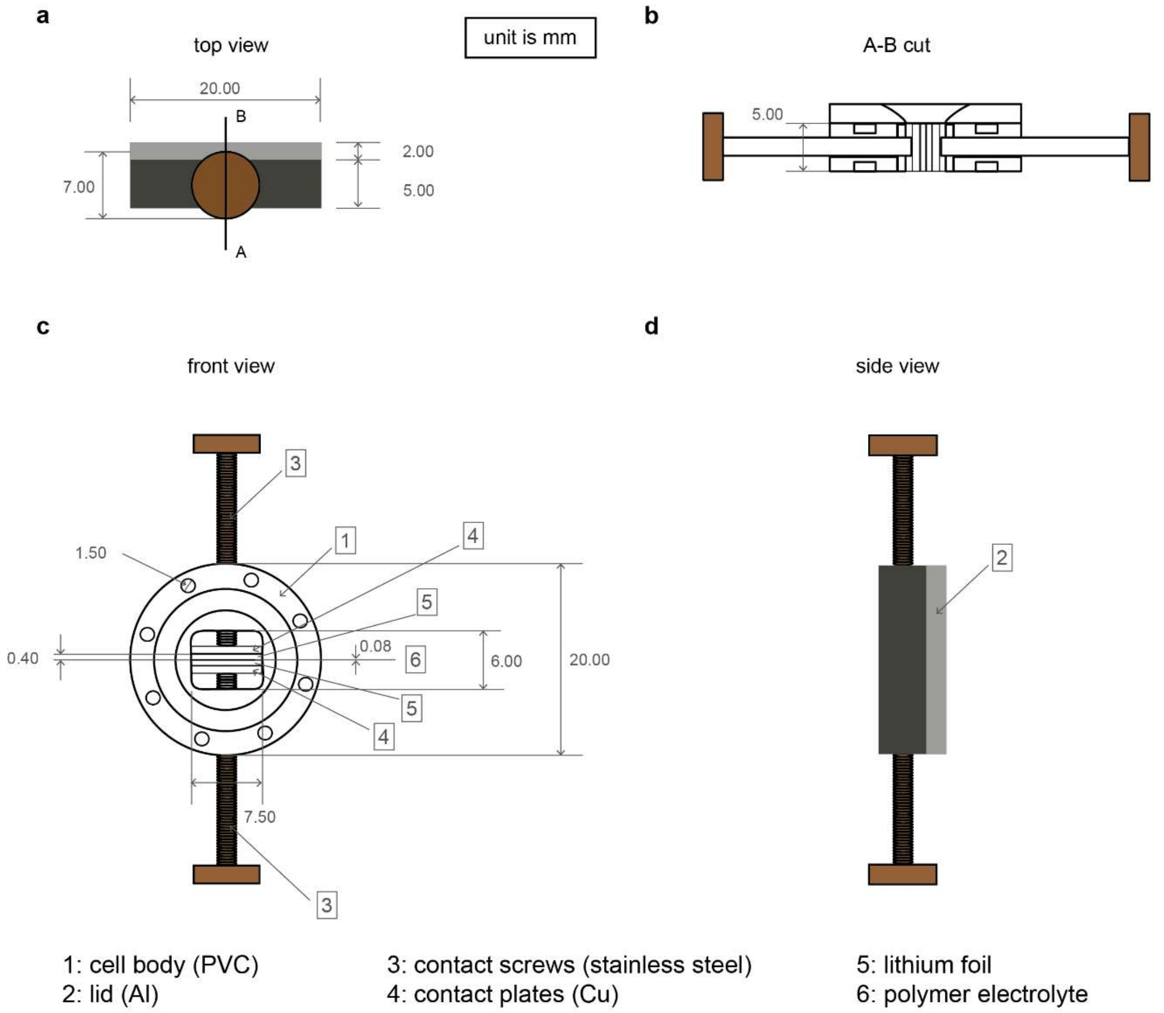


**Supplementary Figure 3.** Construction drawing of the nWAXS cell including the sample.

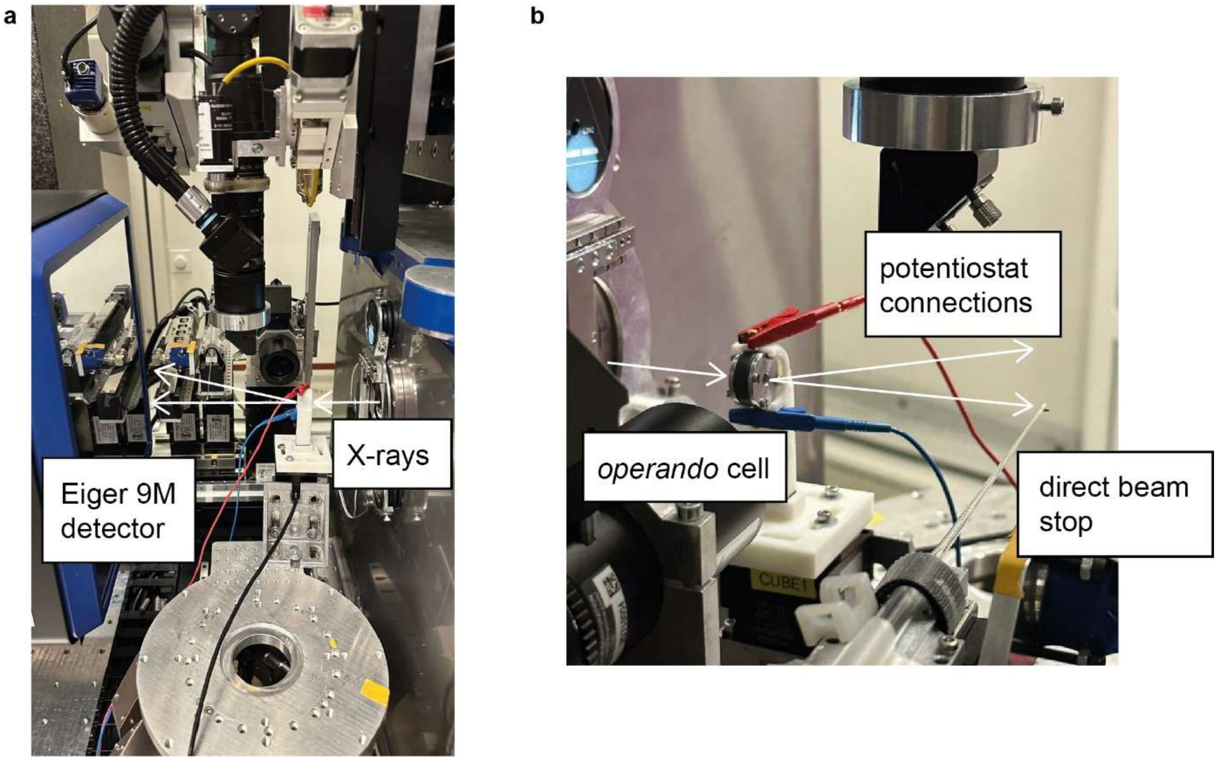


**Supplementary Figure 4**. **a** Experimental setup at the P03 nanofocus endstation at DESY with the X-ray beam coming from the right, passing through the transmission operando cell, and the scattering signal recoded with the Eiger 9M detector. **b** Zoom-in of the set-up to the mounted nano-focus WAXS sample cell with the direct beam stop protecting the detector against the direct beam.

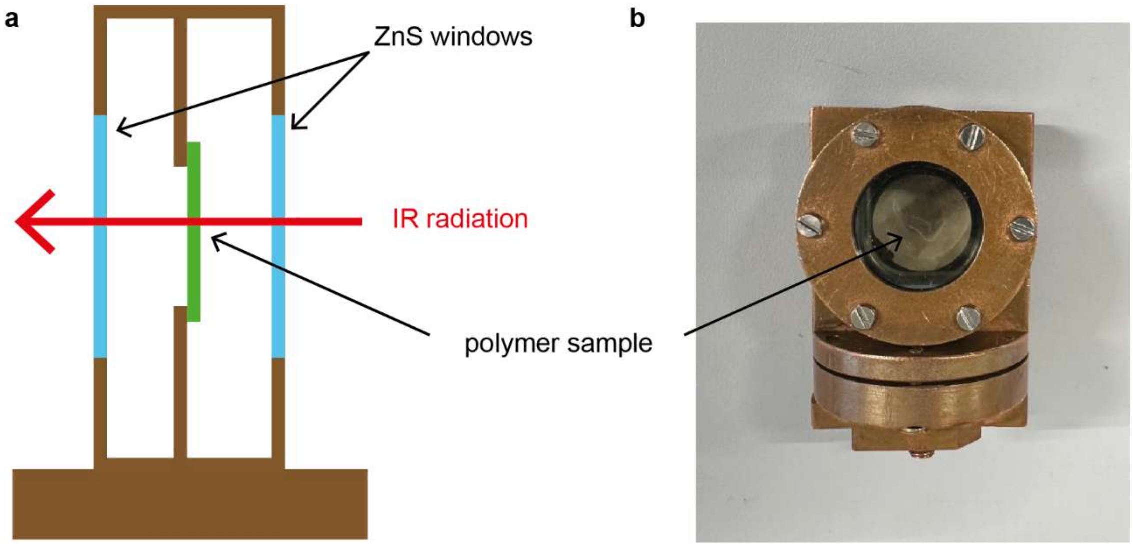


**Supplementary Figure 5.** Sketch and photograph of the FTIR cell.

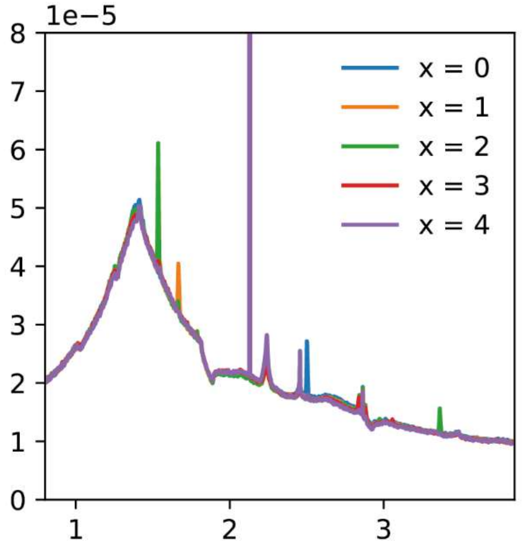


**Supplementary Figure 6.** Peaks at q ~ 1.66 $Å^{-1}$, q ~ 2.84 $Å^{-1}$, q ~ 2.87 $Å^{-1}$, and q ~ 3.36 $Å^{-1}$ can't be clearly identified. Source data are provided as a Source Data file.

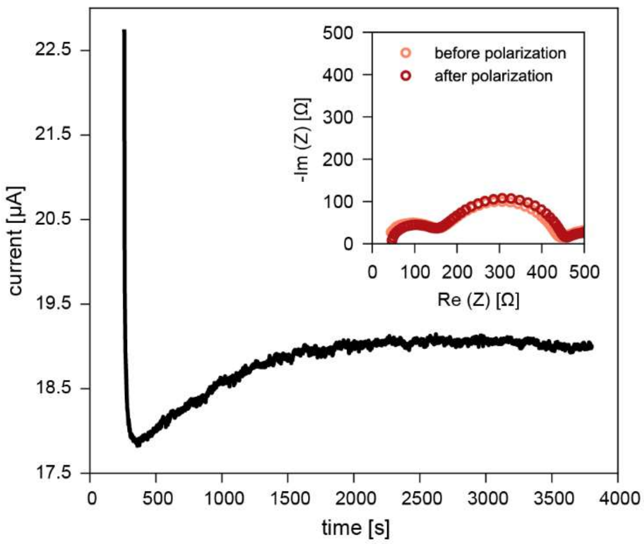


**Supplementary Figure 7**. Transference number test. Source data are provided as Source Data file.
